# New Insights into Cartilage Tissue Engineering: Improvement of Tissue-Scaffold Integration to Enhance Cartilage Regeneration

**DOI:** 10.1155/2022/7638245

**Published:** 2022-01-25

**Authors:** Sahar Jelodari, Amin Ebrahimi Sadrabadi, Fatemeh Zarei, Shahrbanoo Jahangir, Mahmoud Azami, Mohsen Sheykhhasan, Samaneh Hosseini

**Affiliations:** ^1^Department of Tissue Engineering, School of Advanced Technologies in Medicine, Tehran University of Medical Sciences, Tehran, Iran; ^2^Department of Cell Engineering, Cell Science Research Center, Royan Institute for Stem Cell Biology and Technology, ACECR, Tehran, Iran; ^3^Department of Stem Cells and Developmental Biology, Cell Science Research Center, Royan Institute for Stem Cell Biology and Technology, ACECR, Tehran, Iran; ^4^AO Research Institute Davos, Davos, Switzerland; ^5^Research Center for Molecular Medicine, Hamadan University of Medical Sciences, Hamadan, Iran; ^6^Department of Mesenchymal Stem Cells, Academic Center for Education, Culture and Research (ACECR), Qom Branch, Qom, Iran

## Abstract

Distinctive characteristics of articular cartilage such as avascularity and low chondrocyte conversion rate present numerous challenges for orthopedists. Tissue engineering is a novel approach that ameliorates the regeneration process by exploiting the potential of cells, biodegradable materials, and growth factors. However, problems exist with the use of tissue-engineered construct, the most important of which is scaffold-cartilage integration. Recently, many attempts have been made to address this challenge via manipulation of cellular, material, and biomolecular composition of engineered tissue. Hence, in this review, we highlight strategies that facilitate cartilage-scaffold integration. Recent advances in where efficient integration between a scaffold and native cartilage could be achieved are emphasized, in addition to the positive aspects and remaining problems that will drive future research.

## 1. Background

**Figure 1:**
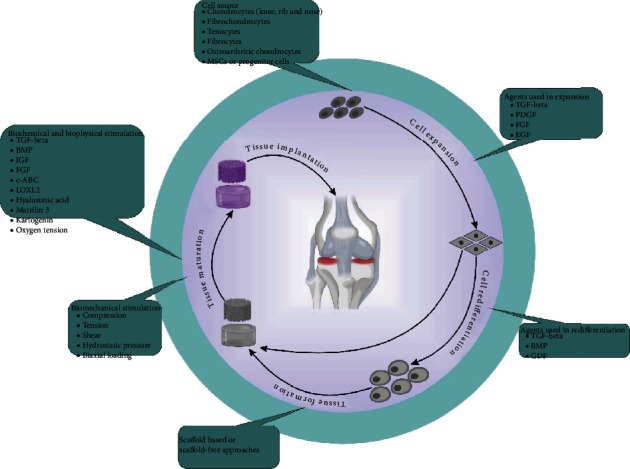
Tissue engineering of cartilaginous tissue. Factors and components like cells, various biochemical, biophysical, and biomechanical stimulations and scaffolds contribute to the development of a well-designed tissue-engineered construct.

Cartilage is a specialized connective tissue with an extracellular matrix (ECM) that is rich in glycosaminoglycans (GAGs) and proteoglycans (PGs). The ECM structure of articular cartilage enables it to reduce pressures and absorb shocks inflicted on the subchondral bone and provides a slippery surface which facilitate bone movement [1, 2]. Articular cartilage exhibits very low intrinsic healing capacity due to its avascular nature and scarcity of cells [3]; therefore, its injury or damage results in pain and loss of mobility in patients, and the need for medical intervention is its inevitable consequence. Despite numerous attempts in pharmaceutical and surgical therapies, they are unable to successfully restore damaged cartilage [4–9]. Cartilage tissue engineering (CTE) has the potential to enhance healing by embedding relevant cells like articular chondrocytes or mesenchymal stem cells (MSCs) and growth factors (GFs) into scaffolds in order to support cell growth and proliferation **(**Figure 1**)** [10–12]. Natural (collagen, gelatin, fibrin, silk fibroin, alginate, hyaluronan, chondroitin sulfate, agarose, and chitosan) and synthetic (polyethylene glycol, poly (lactide-co-glycolic) acid [PLGA], and polycaprolactone [PCL]) biomaterials have been used in CTE [13–15]. However, integration between native cartilage and neoformed tissue remains challenging. Lack of integration can be counterintuitive and lead to further cartilage degeneration [16]. Although suturing or applying adhesives are the current approaches for achieving certain extent of tissue integrity, either of them has its own pitfalls [17]. In this review, we discuss chemical composition, porosity, and load-bearing capacity of biomaterials as properties that can impact scaffold-cartilage integration. Scaffold-free cartilage integration and targeted cell delivery are mentioned as cell-based approaches to repair cartilage defects. Relevant biomolecules in the realm of cartilage regeneration that contribute to seamless integration are discussed. We also explore bioprinting and recruitment of extracellular vesicles as novel strategies for achieving integration between scaffold and native cartilage.

## 2. Designing Scaffolds to Promote CSI

**Figure 2:**
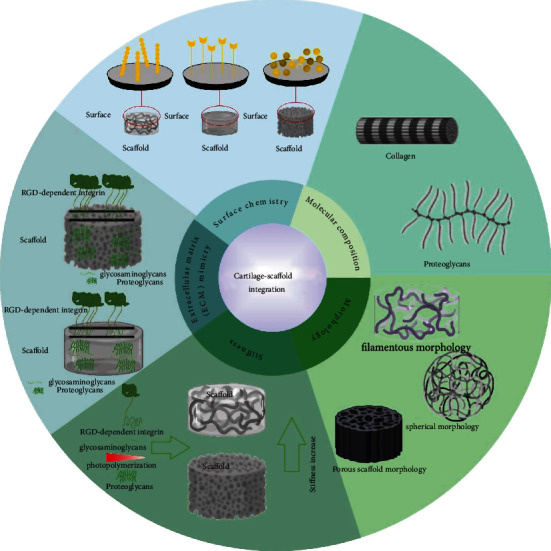
Cartilage-scaffold integration. Several parameters have been marked as significant agents in the CSI.

The characteristics of biomaterials are one of the foremost nonbiological factors that control CSI. A suitable scaffold would provide an optimal and proper architecture for cellular development and attachment. Scaffold properties is an important aspect in tissue regeneration, and resemblance of engineered cartilage to native tissue could enhance integration procedure. To achieve this kind of similarity, different strategies can be developed to manipulate biomaterial's surface chemistry, composition, morphology, and stiffness (Figure 2). For instance, it has been shown that by simulating tissue-like surface chemistry or stiffness, proliferation and growth of chondrocytes or differentiation of stem cells toward desired path can be improved [18–21]. The scaffold's biochemical composition and topographical features can facilitate desirable cell-biomaterial interactions and consequently promote CSI [22, 23]. Below, we describe several features related to CSI that should be taken into consideration in designing and fabricating scaffolds.

### 2.1. Chemical Composition

The chemical composition of scaffolds can alter their physicochemical and mechanical properties and control particular cell/stem cell functions [24]. Researchers have yet to determine whether the ultrastructure or chemical composition of ECM plays a more significant role in stem cell phenotype/lineage determination. Nevertheless, it is crucial to consider the composition of native cartilage in order to establish the proper therapeutic approach. Proteomic analysis of cartilage showed that it is mostly composed of a mixture of highly glycosylated PGs and triple-helical collagens [25], and tensile integrity and remodeling of collagen network were precisely regulated by collagen-interacting matrix molecules. Asanbaeva et al. reported that aggrecan has an inhibitory effect in adherence of collagen fibrils together and hence reduces tensile integrity of immature cartilage [26]. Collagenase delivery by means of nanofibrous scaffold has also been examined. The degenerative capacity of collagenase would result in greater tissue integration [27]. Applying chondroitinase ABC in an in vitro model of cartilage defect also improved integration of self-assembled articular cartilage to native cartilage [28]. Combination of trypsin treatment and delivery of heparin-binding insulin-like growth factor-1 (HB-IGF-1) also stimulates cartilage integration and matrix biosynthesis [29]. Collagen crosslinking agents like lysyl oxidase also facilitated cartilage integration in vitro [30]. Thus, it can be suggested that designing scaffolds to eliminate potential extrafibrillar inhibitory molecules or addition of specific enzymes would reinforce collagen network construction and may result in more robust CSI. However, maintenance of enzymatic activity and controlled release of enzymes would be important under in vivo condition to drive desired results and avoid further tissue degeneration or prolonged inflammation at the same time.

Providing cells with ECM or ECM-derived components also seems promising in optimization of cellular microenvironment and maintenance of viable and proliferating cells which can result in tissue integration. However, it is important to consider the prominent role of different cell types in provoking this integration. It has been shown that combination of decellularized matrices with chondrocytes or other appropriate cell types would promote higher CSI in comparison to decellularized matrix alone [31, 32]. Barthold et al. demonstrated that recruiting ECM microparticles embedded in hyaluronic acid (HA) hydrogel improves chondrocyte migration through chemotaxis and so resulted in tissue integration [33]. It seems that strategies which promote collagen deposition by direct cell recruitment or indirect stimulation of cell migration would result in greater scaffold-cartilage integration.

Endless capacity of biomaterials for developing tissue-adhesive scaffolds could be an attractive regenerative strategy to reach scaffold-cartilage integration [34–42]. In one of the early studies, multifunctional chondroitin sulfate was used as a bridge molecule to enhance attachment of poly (ethylene glycol) diacrylate (PEGDA) hydrogels to surrounding cartilage [17]. This adhesive hydrogel has also been examined in pilot clinical study and was promising when applied to the defect site in combination with microfracture surgery [43]. Photochemical crosslinking is a procedure that relies on rapid and durable bond formation at the scaffold-tissue interface and can cause more robust integration. Arvayo et al. reported the efficiency of phthalocyanine chloride tetrasulfonic acid (CASPc) and aluminum phthalocyanine chloride (AlPc) on articular cartilage integration. Both photosensitizers could augment functional integration without changing cell viability [44]. Using exosome-encapsulated photoinduced imine crosslinking (PIC) hydrogels has led to in vitro and in vivo tissue integration as a result of reaction between aldehyde groups of the hydrogel and amino groups of cartilage [40]. Mohan et al. recruited microspheres to generate material and growth factor gradient which then were used to heal osteochondral defects in rabbit knees. The degree of tissue integration was well beyond the control group [45]. In a recent study, a positively charged elastin-like protein was electrostatically combined with chondroitin sulfate to produce a protein adhesive. This adhesive structure resulted in lateral tissue integration. Better chondrogenesis was also observed due to the release of chondroitin sulfate from electrostatic adhesive [46]. Traditional tissue adhesives may lack some vital aspects of constructive scaffold. These adhesive molecules are not specifically developed to reinforce cellular activities and also may not be suitable to fill large defects [47]. Hence, using adhesive scaffolds could greatly impact integration between scaffold and native cartilage and surpass the effects of traditional tissue glues.

### 2.2. Porosity

Many cellular behaviors such as attachment, cell-scaffold integration, and ECM secretion depend on the porosity and pore size of the scaffold [48]. An appropriate biomechanical and topological environment for cartilage regeneration could be provided via a well-designed porous scaffold. In a study by Matsiko et al., collagen-HA scaffolds seeded by MSCs were manufactured with pore sizes ranging from 94 to 300 microns. Matrix deposition, cell attachment, and chondrogenic gene expression increased in scaffolds with larger pore size [49]. The importance of scaffold porosity has been shown in an in vitro study of meniscus defect repair. In this study, preseeded porous scaffolds resulted in better tissue integration [50]. Porous chondrocyte-seeded honeycomb-like expandable gelatin scaffold is used to repair rabbit femoral condyle defect. Significant tissue regeneration and integration were observed in cell-seeded porous scaffolds in comparison to autologous chondrocyte implantation [51]. Ideal porosity can even lead to satisfactory integration of cell-free scaffolds with native tissue and development of convenient off-the-shelf products [52, 53]. Pore size and density of scaffolds affect ECM integrity and maturity. Interconnected pores increase cell infiltration, ECM secretion, and collagen deposition which consequently could result in better CSI.

### 2.3. Mechanical Loading

Mechanotransduction pathways are fundamental for regulating matrix synthesis in load-bearing cartilage [54]. As a result, adjusted mechanical loading can impact scaffold-cartilage integration. It has been demonstrated that compressive loading augments GAGs and collagen synthesis in cell-seeded hydrogels [55, 56]. However, constructs with poor mechanical properties in comparison to native cartilage can lead to abnormal stress accumulation on the latter part and failure of tissue integration [57, 58] due to altered mechanotransduction signaling. Mechanical stimulation could positively impact tissue integration through increasing collagen synthesis at scaffold-cartilage interface [59]. Yodmuang et al. hypothesized that mechanical discontinuity between scaffold and native cartilage could negatively impact integration. It has been shown that peripheral confinement of cartilage explants prior to compressive loading would increase scaffold-cartilage interface strength. Loading itself also augments GAG content in the scaffolds which follows by more scaffold-cartilage integration [60].

## 3. Recruiting Cells to Achieve CSI

Surrounding dead tissue in the defect edge which has lower cell density can impede integration of grafted tissue to native cartilage. To overcome this hurdle, supplying cells by means of scaffolds could positively affect integration. Since chondrocytes are viable part of the cartilage and responsible for ECM synthesis and henceforth tissue stiffness, their migration is a defining factor in cartilage integration. For example, Src-PLC*γ*1-ERK1/2 signaling pathway affected chondrocyte migration in vitro and inhibition of this pathway resulted in lower interfacial integrative strength [61]. Nevertheless, migration of endogenous chondrocytes to defect site barely occurs in vivo [62]; therefore, cell delivery could be beneficial for superior cartilage integration. Here, we discuss strategies for delivering cellular component to achieve higher scaffold-cartilage integration.

### 3.1. Scaffold-Free Constructs

Cell therapy of cartilage defects is vastly investigated in preclinical and clinical settings [63]. Although the short-term outcomes of these studies are pretty compelling, only patients with minimal or surface cartilage injuries benefit from these therapies in long-term [64]. Park et al. have examined reparative capacity of chondrocyte spheroids and their self-produced ECM in a rabbit model. The constructs exhibited significant ECM accumulation in vitro and excellent integration with surrounding cartilage tissues in vivo [65]. Tuneability, injectability, and biomechanical superiority of human nasal chondrocytes (hNCs) have been confirmed by Gryadunova et al. Injecting hNC spheroids to the explants of bovine intervertebral disc has illustrated its great potential for regeneration of nucleus pulposus (NP) in a minimally invasive procedure [66]. Three dimensional (3D) scaffold-free hyaline cartilage constructs with clinically relevant size were derived from human embryonic stem cells (hESCs). These constructs were mechanically comparable with native cartilage and effectively integrate with human cartilage explants [67]. As mentioned earlier, cells are important components of integrative cartilage repair and their absence would halt tissue integration [31]. Although they are the main contributors to ECM synthesis and cartilage microenvironment maintenance, complete omission of scaffolds from regeneration process may result in poor CSI due to incompatible mechanical properties of native and newly formed cartilage.

### 3.2. Targeted Cell Delivery

Hypothetically, we can assume that targeted delivery of the chondrocytes or stem cells such as MSCs to the injury site would have positive impact on integration between newly formed tissue and native cartilage. The significance of targeted cell delivery (TCD) is accentuated by keeping in mind that tissue regeneration could be easily hampered as a result of cell senescence and death due to anoikis [68, 69]. TCD is achieved by means of different surface functionalization procedures including antibody, peptide, selectin, and genetically mediated modifications which is reviewed elsewhere [70]. Li et al. exploited the targeting of membrane-modified MSCs to the injury site in which the transglutaminase-2 overexpression is a hallmark and utilized as an anchor for modified MSCs. This targeted MSC delivery resulted in better histological scores and gene expression relevant to repair of in vivo cartilage defect [71]. Dual functionalization of PEG by transcyclooctene (TCO) and apoptotic binding peptide (ApoPep-1) led to the development of a crosslinker that binds to apoptotic chondrocytes of injured cartilage explant and methyltetrazine-bearing metabolically active chondrocytes through ApoPep-1 and TCO, respectively. This click chemistry-based pretargeting approach resulted in significant reduction of cartilage degeneration and increased ECM synthesis [72]. Since TCD is a developing approach for repairing cartilage defects, its integrative capacity is not well-studied yet, but is expected to be explored in early future. Theoretically, it can be inferred that precise delivering and attachment of cells to the defect area would have great regenerative impact and can boost ECM synthesis and tissue integration.

## 4. The Role of Biomolecules in CSI

Diverse biomolecules may facilitate integration of native cartilage with the implanted construct (Table 1). Under normal conditions, many regulatory molecules such as growth factors and cytokines act to maintain cartilage tissue homeostasis. These molecules mediate various functions in cell migration, attachment, proliferation, differentiation, and senescence [73]. It is presumed that the ability of cells to migrate towards the defect edge is partially responsible for CSI [74]. However, removal of damaged tissue around the edge of the lesion leaves behind necrotic tissue that acts like a barrier for cellular migration and impedes the desired integration with the host tissue. Chemotactic biomolecules can alleviate this negative condition by enhancing migration of endogenous chondrocytes. In an in vitro study, researchers assessed the chemotactic potential of platelet derived growth factor-bb (PDGF-bb), basic fibroblast growth factor (bFGF), or insulin-like growth factor 1 (IGF-1) combined with an enzymatic rinse to induce cell migration from bovine cartilage explants. It has been shown that brief collagenase treatment followed by supplementation of IGF-1 resulted in significantly increased chondrocyte population around the periphery of the explant [75]. Chemotactic effect of PDGF for chondrocytes was also confirmed in bovine meniscus and PDGF-coated scaffolds resulted in integrative repair of explants [76]. Interestingly, the results of another study confirmed that cartilage integration occurred by inducing chondrocyte migration towards the lesion's edge. Pabbruwe et al. sandwiched a porous bovine collagen scaffold seeded by nasal chondrocytes between two cartilage discs. After 40 days of culture with FGF-2, cell migration between two tissue surfaces across the collagenous membrane induced tissue integration and led to cartilaginous matrix deposition and disappearance of interface borders [77].

Growth factors may promote tissue integration by means of increasing chondrogenic differentiation. Smyth et al. hypothesized that platelet-rich plasma (PRP), as a cocktail of growth factors, might induce graft integration with the host tissue. To evaluate this, osteochondral grafts were treated with either PRP or saline solution before press-fitting into rabbit osteochondral lesions. The graft integration was significantly higher in the PRP-treated group [78]. It was assumed that osteochondral transplantation of MSCs is followed by PRP-induced chondrogenic differentiation. In a recent study, adipose-derived stem cells (ADSCs) were loaded in a thiolated gelatin/poly (ethylene glycol) diacrylate hydrogel with or without IGF-I. The composite hydrogels were then implanted into rabbit osteochondral defects. In contrast to the control group, neocartilage tissue was integrated with the adjacent native tissue in the presence of IGF-I. Incorporation of Coacervates (Coa) resulted in long-term release of IGF-I and even superior tissue integration [79]. Based on this study, the researchers presumed that sustained release of growth factors might provoke CSI. In this regard, Ren et al. fabricated a composite scaffold composed of porcine demineralized bone matrix and poly (alanine ethyl ester-co-glycine ethyl ester) phosphazene (PAGP) microspheres that contained TGF-*β*1, IGF-1, and bone marrow-derived stem cells (BMSCs). Subcutaneous implantation of this construct in nude mice resulted in sustained release of GFs and chondrogenic differentiation of BMSCs with increased GAG deposition compared to the group without GFs. The neocartilage tissue was integrated with the host tissue and exhibited remarkable biomechanical properties that approximated native tissue [80].

Cell recruitment through chemotaxis is another approach to augment tissue integration. Luo et al. used the chemotactic effects of TGF-*β*3 and mechanogrowth factor (MGF) to recruit stem cells at the injury site. Silk fibroin scaffolds were functionalized with either TGF-*β*3 (ST group) or TGF-*β*3/MGF (STM group) and were then implanted into rabbit osteochondral defects. The results showed that STM scaffolds demonstrated superior integration with the host tissue. MGF and TGF-*β*3 synergistically increased endogenous stem cell recruitment. Moreover, MGF downregulated collagen type I expression, which resulted in less fibrocartilage formation and better tissue repair [81].

Recently, peptide biomolecules have been used to recruit cells and promote tissue integration. Bone marrow homing peptide (BMHP) has chemotactic effects on BMSCs homing. Lu et al. utilized BMHP-functionalized acellular matrix-derived scaffolds to enhance cartilage regeneration. The scaffolds were implanted into rabbit full-thickness cartilage defects. Six months postsurgery, defects were filled up with neocartilage tissue that had a smooth surface similar to the native tissue [82]. Lv et al. attempted to integrate the neocartilage with the host tissue by using two custom-designed functionalized self-assembling peptide hydrogels, KLD-12 and KLPP [83]. These peptides possess a functionalized nanostructure with low cytotoxicity that induces chondrocyte and BMSC migration. KLPP has better performance against KLD-12 in vitro. Although, osteochondral defects in the KLD-12 and KLPP groups were suitably filled with neocartilage tissue compared to the control group, and the KLPP group exhibited the most desirable integration with surrounding host tissue. It is concluded that the KLPP peptide hydrogel significantly recruit endogenous chondrocytes and BMSCs and promote tissue integration and cartilage repair.

In brief, growth factors might direct tissue integration by inducing chondrocyte migration towards lesion peripheries, increasing chondrogenic differentiation of stem cells, or recruitment of endogenous stem cells to the area of the lesion. In this context, it is of utmost importance to understand the mechanisms of biomolecule-induced cartilage integration, since any successful regenerative scaffold-based strategy for cartilage repair is contingent upon suitable integration of the construct and the host tissue.

## 5. Novel Strategies for Improvement of CSI

### 5.1. Bioprinting

3D-printing is an intricate method that could improve scaffold integration with surrounding tissue because of the anticipated anisotropic properties of the printed tissue and the possibility of personalized designation of the constructs. In one of the early studies, a poly (ethylene glycol) dimethacrylate (PEGDMA) bioink that contained human chondrocytes firmly attached to the surrounding osteochondral plug after printing. It was hypothesized that direct printing of the hydrogels to the defect site would cause more ECM production and better tissue integration [84]. Shim et al. developed a heterogeneous scaffold by multilayer printing of atelocollagen and supramolecular HA, which was seeded with human turbinate-derived MSCs. They observed cartilaginous tissue formation and integration with adjacent tissues and lack of noticeable inflammation two months postimplantation into rabbit knee joint defects [85]. A layered osteochondral scaffold that consisted of a cartilage layer of gelatin/alginate and a bone layer of gelatin/alginate/hydroxyapatite was seeded with bone marrow stem cells and 3D-bioprinted. It was assumed that effective integration of the printed constructs with native rabbit cartilage was due to the precise biomimetic structure of the scaffolds [86]. In an in vitro study by Daly and Kelly, stratified cartilage structure was achieved due to inkjet bioprinting of cellular spheroids that contained a defined number of MSCs and chondrocytes into preprinted PCL microchambers. This strategy resulted in condensation and organization of cellular spheroids in the PCL microchambers and the formation of an osteochondral construct. This approach facilitated the integration of bone and cartilage regions in these constructs [87]. Gong et al. investigated the effect of bilayered printed scaffolds in repairing an osteochondral injury in a rabbit model. The upper and lower layers were composed of interleukin 4- (IL4-) loaded GelMa and PCL/HA, respectively. IL-4 was used because of its anti-inflammatory properties. There was significantly more tissue integration in the IL4-loaded constructs 16 weeks after implantation compared to the control groups [88]. Recently, Sun et al. reported that 3D-bioprined anisotropic dual-factor (transforming growth factor beta-3 [TGF-*β*3] and bone morphogenetic protein 4 [BMP4] in PLGA microspheres) releasing constructs comprised of PCL and MSC-laden hydrogels led to better cartilage regeneration. These constructs were grafted in knee cartilage defects of a rabbit model. At the six-month follow-up, the researchers noted the similarity of these constructs to normal cartilage in their appearance and gradient structure as well as better microvessel formation that resulted in more robust integration of the graft with surrounding tissue compared to the control group [89]. The regenerative capacity of a functionalized tyramine/methacryloyl gelatin (GelMa-Tyr) bioink was investigated for ex vivo cartilage repair. Unlike most ex vivo studies, the adhesion of printed constructs to surrounding native tissue was evaluated. The presence of tyramine in this hydrogel augmented tissue adhesion by 15-fold compared to GelMa alone [90]. Bioprinting is also utilized to produce an anisotropic construct that benefits from different pore sizes and mimics the gradient structure of native cartilage. Four-layer BMSC-laden bioprinted construct with various pore sizes in each layer has led to better tissue integration and repair in rabbit knee defect [91]. In another study, in situ bioprinting of MSC-laden hyaluronic acid methacrylate- (HAMA-) GelMa bioink via Biopen increased tissue integration in a sheep model of knee cartilage defect [92]. However, there was a brief 8-week follow-up that might prevent conclusions about the efficacy of this procedure. Robotic-assisted in situ 3D-bioprinting of HAMA bioink that was reinforced with an acrylate-terminated 4-armed PEG crosslinker in a rabbit model of cartilage defect was promising in terms of hyaline-like cartilage formation and tissue integration after 12 weeks of follow-up [93]. Most of the abovementioned studies utilized photocrosslinking of the bioinks as the principal procedure for making durable constructs at the defect site. As a result, it can be inferred that this process could be of importance in the future of cartilage bioprinting. In general, the precision of the bioprinting method and, in particular, in situ bioprinting could revolutionize CTE. Additional integrative studies would lead to more chemically designed bioinks and better integration of the implant with native cartilage. The emergence of 4D-printing could be another field of development for CTE. In this approach, 3D-printed constructs would change in accordance with stimulation from the nearby environment. In conclusion, better CSI could be attained with the design of new biomaterials that respond to injured cartilage environment and act suitably to repair the defect.

### 5.2. Extracellular Vesicles

A recent biomolecule-based strategy that included the use of EVs was explored to stimulate cartilage-cartilage integration. EVs are bilayer lipid particles naturally secreted by various types of cells. These secreted membrane-enclosed vesicles range from 10 to 20 nm up to 10 *μ* in diameter and are categorized into exosomes (50-200 nm), microvesicles (MVs) (30-150 nm), and apoptotic bodies (500-1000 nm). EVs mediate intercellular communication and maintain physiological homeostasis through delivery of biological molecules (RNA, DNA, proteins, lipids, metabolites, and some organelles) [94].

EVs have many different adhesion molecules that help them to interact with ECM components and cells. In addition, they can induce the production of fibronectin, collagen type II (COL2A1), and ECM components as well as promote expression of SRY-related HMG-box-9 (Sox-9), proteoglycan 4 (PRG4), aggrecan (ACAN), and cartilage oligomeric matrix protein (or thrombospondin 5) genes [95–100]. EVs can also improve ECM synthesis by reducing the expression of type X collagen (COL10A1) matrix metalloproteinases-1/3/13, runt-related transcription factor 2, aggrecanase-5, and wingless-type MMTV integration site family, member 5A genes [96, 101–104]. These features may lead to improvements in cartilage tissue integration. Moreover, EVs participate in cartilage repair, regeneration, and mineralization processes that eventually promote the integration of cartilage tissue [105].

Another characteristic of EVs that can be attributed to the induction of cartilage integrity is the polarization from M1 to M2 in immune cells, including monocytes and macrophages, as well as promotion of the immune modulation effects by in vitro and in vivo secretion of cytotoxic agents [106]. EVs may be of benefit in the integration of cartilage tissue because of their ability to regulate the AKT and ERK signaling pathways and their capacity to promote the proliferation and infiltration of cartilage tissues [107].

Recent findings suggest that EVs obtained from stem cells may be a promising strategy that could not only ameliorate cartilage healing but also stimulate cartilage integration [40, 108–112]. The results of an animal study on rats with trochlear grooves of both distal femurs showed that exosomes derived from human embryonic MSCs might improve complete cartilage regeneration with good surface arrangement, complete connection to adjacent cartilage, and ECM deposition 12 weeks after surgery, thus ameliorating the probability of cartilage integrity and reducing fibrous tissue formation [107]. In another study, EVs released by BMSCs decreased proinflammatory gene expressions, revoked the TNF-alpha-dependent upregulation of proinflammatory interleukins and COX2, and suppressed TNF-alpha-mediated collagenase activity. They also improved cartilage integration by the promotion of COL II and PG synthesis in osteoarthritis (OA) chondrocytes. Treatment with BMSC EVs led to the suppression of hypertrophic factors gene expressions, such as runt-related transcription factor 2 (RUNX2), type X collagen, and alkaline phosphatase (ALP), which led to an improvement in the cartilage integrity process .

There is evidence that exosomes from BMSCs through kartogenin (KGN) preconditioning (KGN-BMSC-Exosomes) suppress fibrous tissue formation and stimulate the metabolic activity of chondrocytes via promotion of cell proliferation and migration and cartilage matrix production. Thereby, they reduce COL I and c-Myc expressions and, therefore, amplify the probability of chondrocyte maturation and increase COL II, s-GAG, lubricin, and Sox-9 synthesis [99]. With this evidence, KGN-BMSC-exosomes can be impregnated to tissue-engineered scaffolds to improve cartilage repair and integrity. Human umbilical cord-derived mesenchymal stem cell-derived small EVs (hUC-MSCs-sEVs) considerably induced migration, proliferation, and differentiation of chondrocytes. Therefore, they ameliorated the rate of cartilage integrity and increased cartilage tissue regeneration [109]. Additionally, intra-articular administration of EVs secreted by chondrogenic progenitor cells from MRL/MpJ superhealer mice demonstrated a beneficial effect on the proliferation and migration of murine chondrocytes, which might lead to amelioration in the cartilage regeneration [113].

All of the mentioned data show that EVs could be used as suitable candidates for cartilage tissue integrity because of the synthesis of extracellular matrix components, such as fibronectin and collagen type II, as well as their ability to ameliorate the cartilage degradation through inhibition of inflammatory mechanism.

## 6. Concluding Remarks and Future Trends

Despite notable achievements in utilizing novel biomaterials/techniques in CTE, the long-term integration of regenerated hyaline tissues with native cartilage remains elusive and needs urgent consideration. Development of new biomaterials/adhesives to precisely mimic natural cartilage structure and mechanical properties is an undeniable demand for achieving higher order biomechanical integration. On the other hand, apoptosis and necrosis of already rare chondrocytes at the edge of defect is a recurring issue which is partly responsible for lack of proper integration. This hurdle may be overcome by targeted cell delivery to somehow bridging the gap by cell migration and consequently ECM synthesis and metabolism. The precise control of physical or biochemical cues in novel engineered scaffolds could regulate ECM deposition and lead to CSI. The ability to build a native-like gradient of relevant biomolecules can also be beneficial in encouraging cell migration towards the defect edges and result in more robust integration. State-of-the-art in situ 3D-bioprinting is also a promising upcoming trend since it can intricately mimic different aspects of ideal construct. Also, long-term assessments of preclinical and clinical trials could be more informative and ultimately beneficial for resolving integration challenge.

## Figures and Tables

**Table 1:** Biomolecules in cartilage integration.

No.	Recruited biomolecule	Result	Reference
1	FGF-2	Increased GAG and type II collagen biosynthesis. Proliferation and differentiation of chondrocytes	[77, 114–117]
2	IGF-1	Stimulation of proteoglycan synthesis, chondrocyte proliferation, and cell homing. Improved histologic appearance in rabbit full-thickness cartilage defect	[75, 79, 118–123]
3	FGF-18	Increased hyaline-cartilage production	[124, 125]
4	Kartogenin	Intact cartilage regeneration	[126]
5	TGF-*β*1	Improved chondrogenic regeneration and cartilage integrity in a rabbit model	[127]
6	TGF-*β*1/IGF-1	Chondrogenic differentiation, GAG deposition, and neocartilage integration to host tissue	[80]
7	TGF-*β*3	Extracellular matrix formation by fibrochondrocytes of meniscus. Endogenous stem cell recruitment and in situ cartilage regeneration	[81, 128]
8	TGF-*β*3/kartogenin	Promotion of chondrogenesis and cartilage regeneration by synergistic effect and improved integrity in rabbit models	[129]
9	PRP	Enhanced chondrocyte proliferation and redifferentiation. Increase matrix accumulation	[78, 130, 131]
10	BMHP	Stem cell recruitment to the defect site and neocartilage similarity to native tissue	[82]
11	KLPP self-assembling peptide	Recruitment of endogenous chondrocytes and promotion of tissue integration	[83]

BMHP: bone marrow homing peptide.

## Data Availability

Data sharing is not applicable to this article as no datasets were generated or analyzed during the current study.

## Abbreviations

ECM: Extracellular matrix
GAGs: Glycosaminoglycans
PGs: Proteoglycans
CTE: Cartilage tissue engineering
MSCs: Mesenchymal stem cells
GFs: Growth factors
PLGA: Polyethylene glycol, poly (lactide-co-glycolic) acid
PCL: Polycaprolactone
3D: Three-dimensional
EVs: Extracellular vesicles
HA: Hyaluronic acid
HAS: Human serum albumin
PLL: Poly-L-lysine
CASPc: Phthalocyanine chloride tetrasulfonic acid
AlPc: Aluminum phthalocyanine chloride
PEGDMA: Poly (ethylene glycol) dimethacrylate
IL4: Interleukin 4
TGF-*β*3: Transforming growth factor beta-3
BMP4: Bone morphogenetic protein 4
GelMa-Tyr: Tyramine/methacryloyl gelatin
HAMA: Hyaluronic acid methacrylate
PDGF-bb: Platelet-derived growth factor-bb
bFGF: Basic fibroblast growth factor
IGF-1: Insulin-like growth factor 1
FGF2: Fibroblast growth factor 2
PRP: Platelet-rich plasma
ADSCs: Adipose-derived stem cells
COL II: Collagen type II
PAGP: Poly (alanine ethyl ester-co-glycine ethyl ester) phosphazene
BMSCs: Bone marrow-derived stem cells
MGF: Mechano growth factor
BMHP: Bone marrow homing peptide
LPP: Link protein N-terminal peptide
ICRS: International cartilage repair society
MVs: Microvesicles
OA: Osteoarthritis
RUNX2: Runt-related transcription factor 2
ALP: Alkaline phosphatase
KGN: Kartogenin
hUC-MSCs-sEVs: Human umbilical cord-derived mesenchymal stem cell-derived small EVs.
